# Electret integrated magnetic field sensor based on magnetostrictive polymer composite with nT resolution

**DOI:** 10.1038/s41598-024-85069-6

**Published:** 2025-01-10

**Authors:** Lukas Zimoch, Stefan Schröder, Eric Elzenheimer, Sören Kaps, Thomas Strunskus, Franz Faupel, Michael Höft, Rainer Adelung

**Affiliations:** 1 Functional Nanomaterials, Department of Materials Science, Kiel University, Kaiserstr. 2, 24143 Kiel, Germany; 2 Chair for Multicomponent Materials, Department of Materials Science, Kiel University, Kaiserstr. 2, 24143 Kiel, Germany; 3 Chair of Microwave Engineering, Department of Electrical and Information Engineering, Kiel University, Kaiserstr. 2, 24143 Kiel, Germany

**Keywords:** Magnetostrictive polymer composite (MPC), Nanoscale sculptured aluminum, Electret, Magnetic field sensor, Sensor evaluation, Electronic devices, Sensors and biosensors, Electronic properties and materials, Magnetic properties and materials

## Abstract

The pursuit for advanced magnetoelectric field sensors has gained momentum, driven by applications in various fields, ranging from biomedical applications to soft robotics and the automotive sector. In this context, a capacitive read-out based magnetostrictive polymer composite (MPC) sensor element is introduced, offering a new perspective on magnetic field detection. The sensor element’s unique feature is the possibility to independently tailor its mechanical and magnetic properties. When compared to other composite-based magnetic field sensors, the limit of detection (LoD) is three orders of magnitude lower (95.6 nT/√Hz at a resonance frequency of 160.5 Hz). In contrast to other electret-based ME sensors, the LoD is reduced by a factor of 20. To the best of the authors’ knowledge, this work marks the first comprehensive attempt to characterize a sensor magnetically, aligning with the thorough assessment standards of ME sensors. This study aims to narrow the disparity between established magnetic field sensors, featuring consistent characterization protocols, and the novel MPC sensors, which often undergo limited magnetic characterization as part of their evaluation. The presented sensor, built from readily available materials, offers a versatile and tuneable platform for magnetic field detection, and ongoing research aims to unlock its full potential in diverse applications.

**Supplementary Information:**

The online version contains supplementary material available at 10.1038/s41598-024-85069-6.

## Introduction

The market for magnetoelectric field sensors is experiencing growth due to increasing demand in various industries, such as the automotive sector and especially in biomedical applications for systems with navigation, orientation, and motion tracking. However, they can also be utilized for electronic skins, smart textiles, soft robotics and actuators^1^.

The most advanced sensors that are capable of detecting magnetic signals as small as the signals of the human brain are superconducting quantum interference devices (SQUIDs)^2,3^. Due to their bulky size and requirement for cryogenic cooling, they result in significant costs, making their widespread use challenging^4,5^. An alternative to SQUIDs are optically pumped magnetometers (OPMs), which achieve performances similar to those of SQUIDs without the need for cryogenic cooling^6,7^. Sensor such as fluxgate magnetometers or Hall effect sensors are limited by magnetic noise and their sensitivity cannot be reduced further^8^.

Another common approach for magnetoelectric field sensors is to utilize a combination of a magnetostrictive material, which is sensitive to magnetic fields, and a mechanically linked piezoelectric material, which generates an electrical signal proportional to the magnetic field of interest. These sensors are usually referred as Magnetoelectric (ME) sensors^9,10^.

An alternative method involves the integration of a Fiber Bragg grating (FBG) with a magnetostrictive cantilever. The deformation of the FBG modifies its reflection spectrum, which can be related to the magnetic signal^11,12^.

Cantilever-based sensors face limitations in adjusting mechanical properties independently from the magnetic properties. Cantilever-based sensors have the drawback, that it is not easy to freely change the mechanical properties without changing the magnetic properties. Attempts were undertaken by utilizing an electret to manipulate the resonance frequency of the cantilever resulting in an undamping, which can be related to the electrostatic interactions^13^. The sensor presented in this work uses a magnetostrictive polymer composite, which consists of ferromagnetic particles embedded in a polymer matrix. The mechanical properties are defined by the matrix material, which can be tuned or totally replaced by a different polymer. This gives the opportunity to change the resonance frequency of the sensor without changing the geometry, or to protect the magnetic particles from environmental conditions such as corrosion. On the other hand, the type or number of magnetic particles can be changed. This metamaterial emerges as a compelling candidate for magnetic field sensing, particularly due to its unmatched stretching capabilities under magnetic field exposure. In the MPC no remagnetization takes place. Due to the soft polymer matrix the particles themselves rotate and move, causing a similar effect than in a magnetostrictive material.

Numerous studies have investigated MPC-based magnetic field sensors by subjecting cantilevers to significant magnetic fields in the mT range. These studies detect cantilever bending through methods such as image processing^14^, monitoring shifts in Bragg wavelength via FBG^11,15^, or by performing magnetoimpedance and evaluating electrical resistance changes^16^.

While these investigations thoroughly explore the mechanical and magnetic properties of emerging sensor concepts, comparing measurements across different sensors presents challenges due to their diverse characterization methods. It is also crucial to evaluate if the sensor responses are linear with variations in magnetic field strengths. While some literature defines low magnetic fields as those under 1 T^17^, relevant biomedical applications like magnetocardiography (MCG) or magnetoencephalography (MEG) require detecting field amplitudes in the pT or even fT range, which are at least 12 orders of magnitude smaller. Claims of measuring fields in the mT range down to 0 mT can be misleading, suggesting the ability to detect any magnetic field. For ME sensors Elzenheimer et al.^18^ proposed a sophisticated evaluation scheme, which has been applied to the MPC-based sensor of this study. Sensors from various categories require consistent evaluation methods to enable meaningful comparisons, even in the field of highly sensitive magnetometers.

The sensors can be fabricated in complex shapes by designing molds beforehand, but it is also possible to cut the sensor mechanically or with a laser after the fabrication.

The MPC is electrically contacted with a thin nanoscale sculptured aluminum^19^ sheet. This sculpturing does not affect bulk properties like conductivity but leaves a rough surface with a hook like structure. This allows a stable mechanical connection to the chemically inert silicone. The contacted composite is clamped on one end and oriented parallel to an electret. The electret has quasi-permanent surface charges *Q*^20^. Both components form a structure, which can be interpreted as a plate capacitor (cf. Equations 1 and 2). When an AC magnetic field is applied, the MPC begins to oscillate, which alters the distance *d* between the two components and subsequently the capacity *C*:

1 $$\:C\:=\:{\epsilon\:}_{0}{\epsilon\:}_{r}\:\bullet\:\:\frac{A}{d}$$

2 $$\:U\:=\:\frac{Q}{C}$$

where *ε*_*0*_ is the vacuum permittivity, *ε*_*r*_ is the relative permittivity, *A* is the area of the two plates and *U* is the voltage signal of the sensor. Because the charges are constant, due to the nature of the electret, the voltage signal is only changed by the displacement of the cantilever. In recent studies, researchers have explored a rudimentary form of magnetostrictive polymer composites consisting of two permanent magnets stacked atop one another^21^. The upper magnet is attached to a silicon cantilever with a piezoelectric thin-film layer. When an external magnetic field is applied, it interacts with the magnetic field between the two magnets. This setup provides insight into the particle interaction within the composite. The magnetic interaction among the particles is affected by the external field, resulting in an effect similar to that observed in magnetostrictive materials.

In this study, we employed materials that are readily accessible and cost-effective, including silicone (PDMS), carbonyl iron particles and bulk Teflon. The silicone encapsulates the magnetic particles making the cantilever resistant to harsh environmental conditions due to the chemically inertness of PDMS^22^. The silicone used in this work is also biocompatible making it favorable for biomedical applications.

Although the sensor performance is not at the state-of-the-art level in terms of sensitivity and limit of detection values, it presents numerous tuning possibilities, and several parameters still need to be optimized. Unlike some other sensors, there is no need for an initialization step to adjust the magnetic domain structure^23^. In contrast to MPC-based sensors, the attained Limit-of-Detection of 95.6 nT/√Hz represents a substantial advancement^24^.

## Results

**Fig. 1:**
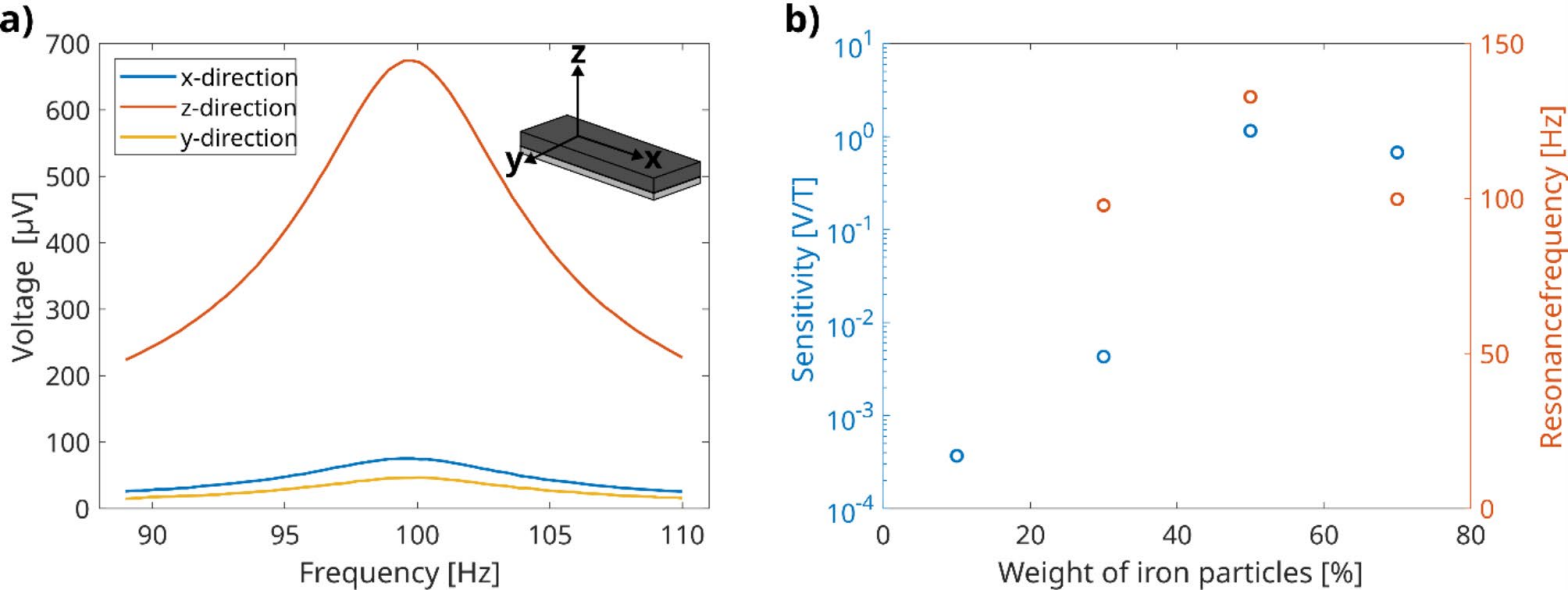
Vector behavior and influence of particle concentration. (**a**) The first plot shows the resonance behavior with 70 wt% iron particles. The relative orientation to the applied magnetic field has a strong influence on the absolute signal amplitude. The sensor is only sensitive in the z-direction. (**b**) Compares sensor elements with different magnetic particle concentrations. The signal increases up to 50 wt% and finally decreases at a concentration of 70 wt%.

As described in “Sensor assembly”, the outer frame of the housing of the sensor element is cube like, which gives the option to orient the sensor in the aluminum tube and measure along all three spatial directions. The size of the cube also prevents it from rotating in the tube. Figure 1 shows the results. By applying the magnetic field along the cantilever direction, which is defined as the x-axis (cf. inset of Fig. 1), the sensor generates a signal, which shows a pronounced resonant behavior. By applying the magnetic field along the z direction, the signal is increased by a factor of 10. If the magnetic field is applied along the y direction, the signal magnitude is in the same order of magnitude as in the first case. All consequent measurements were performed along the orientation with the highest signal.

The magnetic particle concentration has also an impact on the sensitivity as well. It is given in weight percentages (wt%) and always refers to the amount of iron particles in comparison to the amount of silicone. Four different sensor elements were investigated, ranging from 10 wt% to 70 wt%. The different resonance frequencies and resulting sensitivity responses are depicted in Fig. 1. The sensor element containing 10 wt% iron particles cannot detect the applied magnetic fields. The given value for the sensitivity is the average of all the data points of the frequency sweep (cf. supplementary figure S1). While the 30 wt% sensor demonstrates the capability to register the applied magnetic field, the resulting output signal remains comparatively modest with 4.3 µV and the frequency sweep is dominated by noise. The most substantial signal, measuring 1.15 mV, is generated by the sensor employing a particle concentration of 50 wt%. However, a further increase of the concentration, up to 70 wt%, leads to a reduction in the signal magnitude, which diminishes to 0.67 mV.

As described in “Magnetic field measurements”, a minor symmetric hysteresis loop measurement was performed in the range from − 1 mT to 1 mT. This finding verifies that the magnetization of the sensor itself does not change during the magnetic measurements. The largest sensor signal is reached at a static DC bias field of -533 µT (cf. supplementary figure S3). Thus, the following measurements were performed with this bias field.

**Fig. 2:**
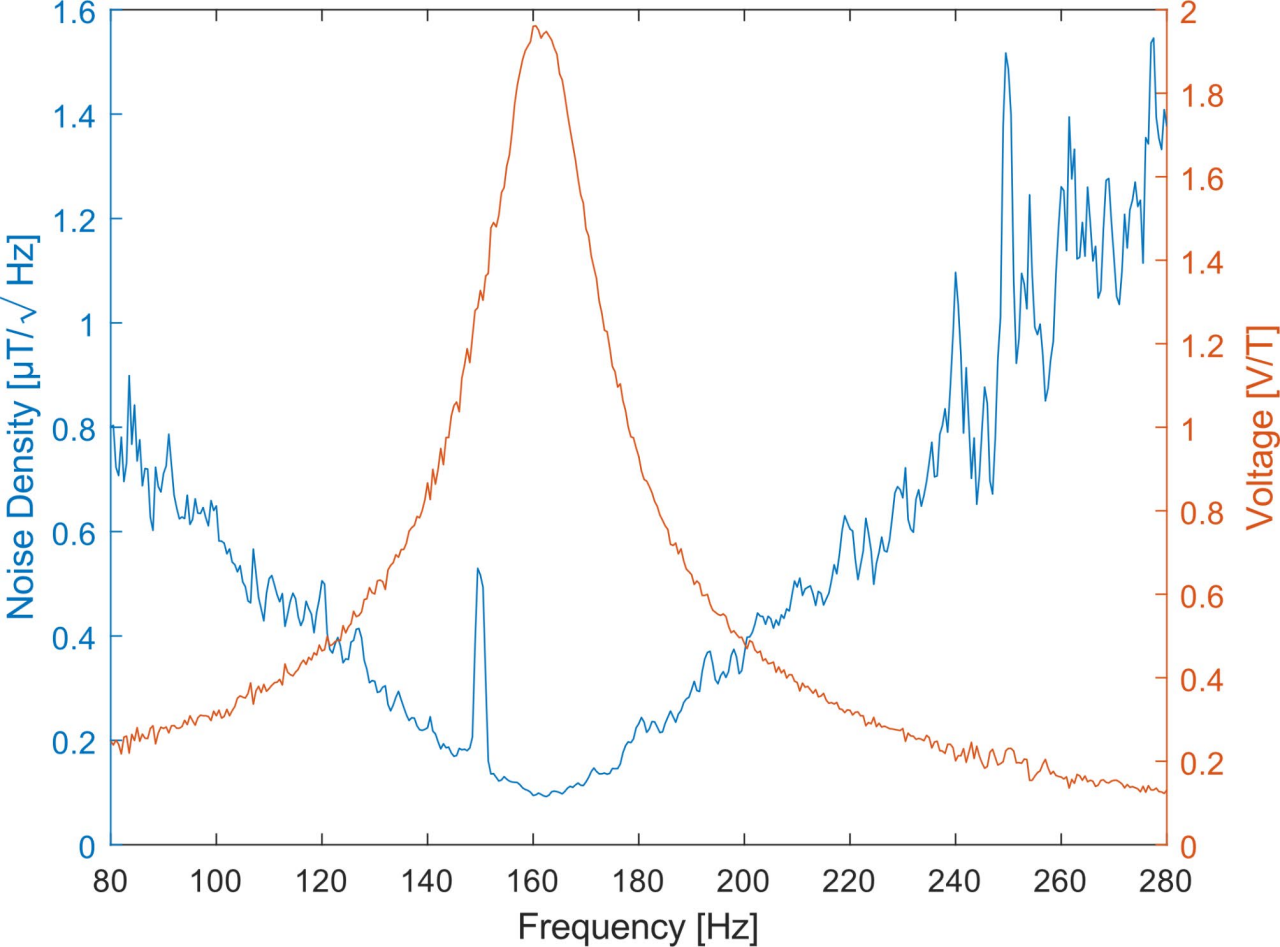
Performance of the sensor with 50 wt% iron particles. The red line shows the amplitude behavior of a typical sinusoidal frequency sweep of the investigated sensor. The resonance frequency is at 160.5 Hz and has a sensitivity of 1.96 V/T. The blue curve depicts the equalized noise density spectrum, indicating a lowest value of 95.6 nT/**√**Hz in resonance.

As described in  “Magnetic field measurements”, a frequency sweep was performed between 80 Hz and 280 Hz, which reveals a mechanical resonance frequency of 160.5 Hz. This is the point where the sensor is most sensitive and thus detects the smallest magnetic fields (cf. Figure 2). The equalized spectral noise density has its minimum at the resonance frequency. A higher or lower frequency results in a decrease in the signal-to-noise ratio (SNR), due to the lower sensitivity of the sensor. The quality factor is 8, which leads to a 3-dB-Bandwidth of approximately 20 Hz. At resonance the sensitivity is 1.96 V/T. The noise amplitude spectral density reached at resonance is 95.6 nT/√Hz.

It is misleading to chase for higher and higher sensitivities. This is only favorable if the noise is not increased in the same way. Schoinas et al. reported on a sensor with a high sensitivity of 14,620 V/T^25^. The lowest detectable field is at ± 40 µT. Like the sensor presented in this work, it is cantilever based. Even though the sensitivity is four orders of magnitude lower (1.96 V/T), this works sensor is capable of detecting fields that are three orders of magnitude smaller (95.6 nT/√Hz).

## Discussion

The results show that sensor elements require high particle concentrations to have large sensitivities. When the concentration is too low, the particles interact like individual units and do not influence each other due to the large distance between them. With increasing concentration, the particles in the composite are closer together, which increases the interaction between them and thus, the deflection of the cantilever is more pronounced. There seems to be a maximum distortion of the cantilever at around 50 wt%, potentially at slightly higher concentrations. This gives the opportunity to increase the signal further just by tweaking the particle concentration. At 70 wt% the amplitude of the signal is already decreased. By increasing the particle concentration, the composite’s stiffness increases (cf. figure S4), as the mechanical properties of the iron particles become more prominent. An optimal point is reached where the composite contains a sufficient quantity of magnetic particles to induce maximal deformation in the polymer matrix, while avoiding an excessive level that would compromise the flexibility of the matrix.

When the sensor element is exposed to a magnetic field, several interactions occur between the particles. The particles act slightly different on the magnetic field, because they are not perfectly aligned, they change their position towards each other and the particles rotate. Among these interactions, particle rotation emerges as the most influential factor. The polymer chains around the particles are distorted, causing significant deformations in the surrounding matrix and consequently leading to substantial cantilever deflection. The orientation of the magnetic active material plays a crucial role and leads to a true vector sensor, which is sensitive in only one direction. The particles are oriented along the long axis, denoted as the x-axis (cf. Figure 1), of the cantilever. When a magnetic field is applied in the x-direction, the particle rotation remains limited, resulting in a relatively weak signal response. Similarly, when measurements are conducted along the y-axis, the signal strength remains comparatively modest. In this scenario, particle rotation is feasible but constrained by the mechanical rigidity of the aluminum contact. The contact exhibits high stiffness, preventing in-plane deformation induced by the rotating particles. When the field is applied in the z-direction the signal increases by a factor of 10. Similar to the case of the y-direction the particles can rotate, leading to an out-of-plane deformation of the aluminum contact that requires considerably less energy. As a result, this leads to a more pronounced deflection, which is directly proportional to the signal amplitude.

The bias measurement shows a constant signal for fields larger than ± 600 µT. This can be explained by the strong electrostatic interaction of the sensor components. The electret is negatively charged and creates positive mirror charges at the electrode below the cantilever. When these two components come closer together even more charges are created. Simultaneously the attractive forces between the positive and negative charges increase drastically. The cantilever snaps to the electret and can only move partially due to the strong attractive forces, thus resulting in a smaller signal.

## Conclusion and outlook

The novel magnetostrictive polymer composite sensor presented in this study offers promising possibilities for magnetic field detection. By employing silicone as the polymeric matrix and integrating ferromagnetic iron particles, this sensor demonstrates tunability in mechanical and magnetic properties, providing a versatile platform for optimization. The MPC is effectively contacted using nanoscale sculptured aluminum, ensuring a robust and reliable electrical connection and utilizes an electret, which provides a permanent electric field for the read-out.

The sensor’s resonance frequency, at 160.5 Hz, offers sensitivity to small magnetic fields, with a limit of detection as low as 95.6 nT. This is a big improvement compared to the existing composite-based and electret-based sensors, resulting in a lower LoD of three orders of magnitude and a factor of 20 respectively. Its 3-dB-Bandwidth of 20 Hz and quality factor of 8 suggest a small but sensitive frequency range.

Increasing the particle concentration to around 50 wt% leads to a higher sensitivity. The sensor element has a predominant direction making it suitable for detecting the direction of a magnetic field by combining several elements. It can also reduce directional magnetic noise, by orienting it unfavorable for the noise. The sensor is built by broadly available materials such as silicone, iron, PTFE and aluminum. The manufacturing process is straightforward and allows a tremendous number of tuning possibilities before, during and after the sensor element preparation. By choosing silicone as polymer, the sensor can withstand harsh environmental conditions and is biocompatible.

Future improvements may involve the substitution of iron particles with harder magnetic materials, such as Neodymium-Iron-Boron alloys, to reduce noise originating from the magnetic component. Optimization of the aluminum contact and exploration of different geometries could further enhance the sensitivity.

## Methods

### Magnetostrictive polymer composite fabrication

The magnetostrictive polymer composite was fabricated using two-component silicone (Ecoflex 00–30, Smooth-On, PDMS, 20 g). The components were mixed with carbonyl iron particles (Sigma, diameter: 20–100 μm, 20 g) in a paper cup. During the mixing process, air entered the mixture and was removed by subjecting the dispersion to a low-pressure environment in a custom-built desiccator connected to a scroll pump. The pressure was reduced to 1 mbar within the first 30 s and was maintained for an additional 5 min to eliminate air bubbles from the composite. Subsequently, the desiccator was flooded with air until ambient pressure was reached.

The MPC fabrication involved the use of a nanoscale sculptured aluminum sheet (cf. Chapter 5.2) with a thickness of 100 μm as the bottom layer, with the sculptured surface facing upwards, in a 3D-printed polylactic acid (PLA) mold with an inner diameter of 25 mm × 25 mm × 1 mm. The liquid MPC was poured on top of the aluminum sheet and a polymethyl methacrylate (PMMA) plate was placed on top of it to eliminate excess composite and create a smooth, flat surface. While the silicone is liquid, it fills the cavities of the aluminum. The silicone started to become viscous after 30 min and fully cured after 4 h at room temperature. To orient the particles the curing takes place in a magnetic DC field of 100 mT. In contrast to magnetostrictive materials no magnetic moments have to be aligned in an annealing process and the magnetization of the individual particles does not change during the operation of the sensor within the range of the tested bias fields (cf. figure S3).

Due to the hook like structure, the cured MPC is mechanically connected to the aluminum, which serves as the read-out electrode of the sensor. The high viscosity of the MPC prevented the iron particles from fully sedimenting, and the composite can be cut into various shapes using mechanical or laser cutting techniques after curing. The cantilevers of the sensors have a size of 12 mm x 5 mm x 1 mm.

### Electrical contact

The establishment of a reliable electrical readout-contact with the MPC presents certain challenges, primarily attributed to the intrinsic properties of silicone. In an initial attempt, the application of silver glue from Acheson, specifically Silver DAG 1415, was explored using a brush. While this approach initially facilitated wetting of the MPC, it caused issues such as delamination during the drying process and deformation of the composite. Subsequently, a 200 nm-thick gold film was investigated, which was deposited through physical vapor deposition. However, this method proved to be less conductive than desired, potentially due to the rough surface topography arising from the 3D printed molds used in the fabrication process.

To establish a contact that is both reliable and durable, nanoscale sculptured aluminum was considered. In this approach, 100 μm-thick aluminum sheets underwent an etching process employing hydrochloric acid (HCl) from one side. The HCl solution was prepared at a concentration of 7.25 wt%, and etching was carried out at room temperature without stirring. This procedure yielded a distinctive hook-like structure comprised of cuboids, oriented at 90° angles to each other. This etching technique exhibited no preferential grain boundary dissolution, but it is defined by the crystallographic orientation of the grains. Specifically, the {100} plane is more stable, resulting in flat surfaces at both nanometer and micrometer scales^19^.

### Electret fabrication

The electrets utilized in the current sensors are made from 1 mm thick bulk Polytetrafluoroethylene (PTFE or Teflon) with dimensions of 12 mm × 12 mm, which were obtained from a larger sheet. A self-built corona discharge setup^20^ was utilized to impart surface charges on the PTFE. The setup consisted of a metal sheet serving as the ground electrode, with the PTFE placed atop it and a wire serving as the discharge electrode. A voltage of 5 kV was applied for 1 min. It is important to allow the electret to rest for at least one day prior to use in the sensor to ensure that any shallow charges that may contribute to sensor noise and negatively impact the limit of detection have dissipated.

### Sensor assembly

**Fig. 3:**
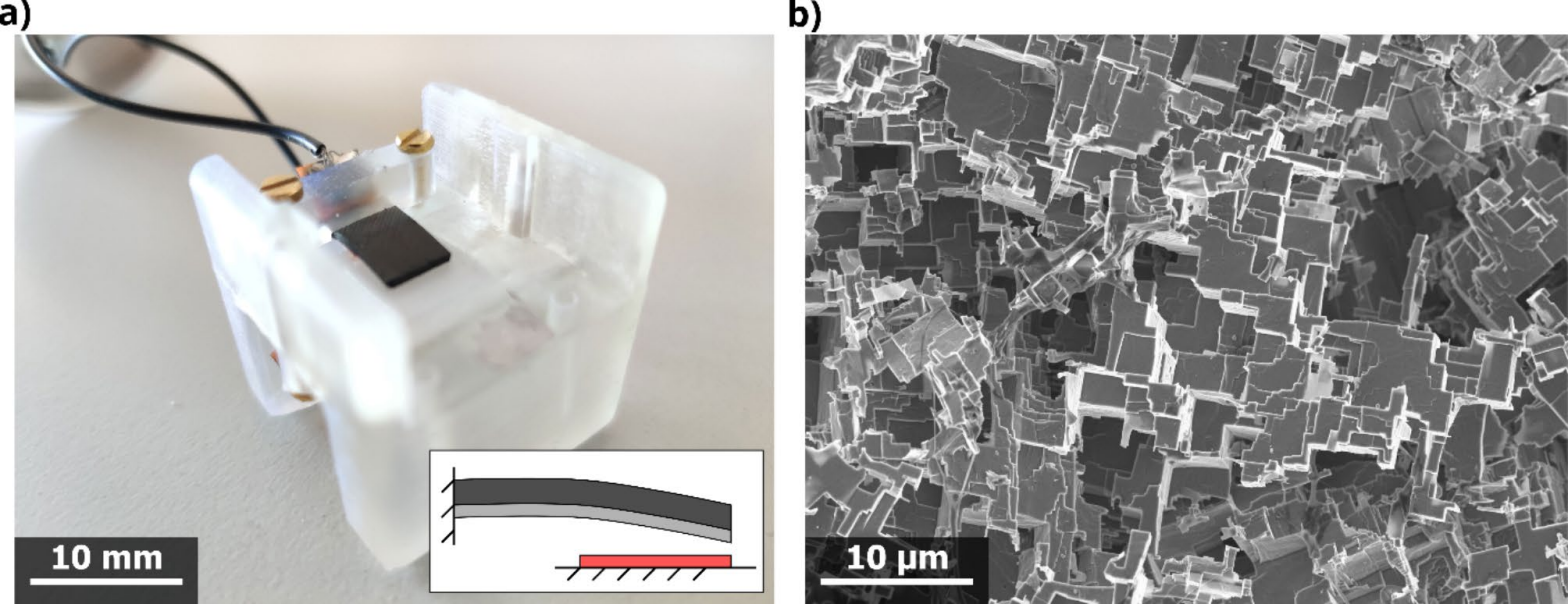
Assembled sensor with details. (**a**) Sample holder. The composite (gray) is clamped and slightly bent to the electret, which is located directly below (shown in red in the schematic inset). (**b**) SEM image of nanoscale sculptured aluminum. Liquid silicone can fill the cavities, which leads to a mechanical interlocking.

The sensor housing (cf. Figure 3) was fabricated using a “Form 2” 3D printer from Formlabs. The Clear V4 resin was used and the part was printed at a resolution of 50 micrometers. The cube-shaped design features clamps for securing the electret on the bottom and the cantilever on the top, with the option of using a double-clamped cantilever for various excitation modes. Its size and shape allow for insertion into a coil pair used to generate magnetic fields for the measurements, but it cannot rotate once inserted. Measurements along all three directions (cf. Chapter 2 and Fig. 1) in space can be performed. A copper sheet with a cable soldered to it is attached to the cube, serving as the sensing contact for picking up the generated signal by the MPC. A separate copper contact is positioned near the MPC and used as a reference, enabling differential sensor readout, and reducing overall noise.

### Magnetic field measurements

The magnetic field measurements were performed with a custom-made setup, which is placed on a vibration-proof table. A coil pair is used to generate AC and DC magnetic fields. The DC-coil has approximately 1500 windings and is used to generate a magnetic bias field to operate the sensor at the optimal working point. The AC-coil has around 750 windings and creates the desired AC magnetic field, which can be detected by the sensor. Both coils have a length of 15 cm. Everything is in a metal box to shield the sensor from electrical noise coming from the environment. In addition, a thin aluminum tube is inserted into the coils for additional electrical shielding. Both parts are connected to the ground. The coils are wrapped in 5 isolated layers of Mu-Metal with a thickness of 300 μm to shield the sensor from magnetic noise.

The sensor has a mechanical resonance frequency at which it is most sensitive. This is determined by a sinusoidal frequency sweep in steps of 2 Hz, followed by a high-resolution measurement to determine the exact frequency^18^. During these measurements, a constant field strength of 100 µT is used. Next, a DC bias field measurement is conducted. This involves applying a constant AC field with a frequency matching to the resonance frequency of the sensor, with an amplitude of 100 µT. A static DC field ranging from − 1 mT to 1 mT is then applied in both directions, in order to ensure that the magnetization of the sensor remains unchanged. The optimum working point is identified as the point at which the sensor produces the highest signal. Finally, the smallest detectable field is determined through a series of measurements (cf. Figure 2S) in which the sensor is operated under ideal bias field conditions and in resonance. During each measurement, the AC magnetic field is gradually reduced. At one point, the generated AC magnetic field reaches the sensor’s noise level. At a certain point, the generated AC magnetic field approaches the sensor’s noise level. At this juncture, the noise power produced by the sensor surpasses the signal power of the generated magnetic field.

## Electronic supplementary material

Below is the link to the electronic supplementary material.

Supplementary Material 1

## Data Availability

All relevant data are available from the corresponding author Rainer Adelung, ra@tf.uni-kiel.de upon reasonable request. Any additional information required to replicate the results of this study is also available from the corresponding author upon reasonable request.
