# Supplementary material for: Electret integrated magnetic field sensor based on magnetostrictive polymer composite with nT resolution
Source: Sci Rep. 2025 Jan 10;15:1561. doi: 10.1038/s41598-024-85069-6 (PMC11724109; doi:10.1038/s41598-024-85069-6)
Supplement: Supplementary file 1 — Supplementary Material 1 [file 41598_2024_85069_MOESM1_ESM.docx]

**Supplementary**

Electret Integrated Magnetic Field Sensor Based on Magnetostrictive Polymer Composite with nT Resolution

Authors:

Lukas Zimoch^*,1^, Stefan Schröder^2^, Eric Elzenheimer^3^, Sören Kaps^1^, Thomas Strunskus^2^, Franz Faupel^2^, Michael Höft^3^, Rainer Adelung^*, 1^

* Corresponding authors, E-Mail: luzi@tf.uni-kiel.de, ra@tf.uni-kiel.de

Figure S1 shows frequency sweeps from four sensor elements, with a different filling factor of iron particles. The sensor element containing 10 wt.% iron particles is not able to detect the applied magnetic fields. A closer examination, as depicted in the inset, reveals the signal of the sensor element containing 30 wt.% of iron particles. The resonance frequency at 97 Hz has a signal strength of about 4 µV. The largest signal is generated by the 50 wt.% sensor element, while the signal decreases by increasing the particle concentration further, up to 70 wt.%.


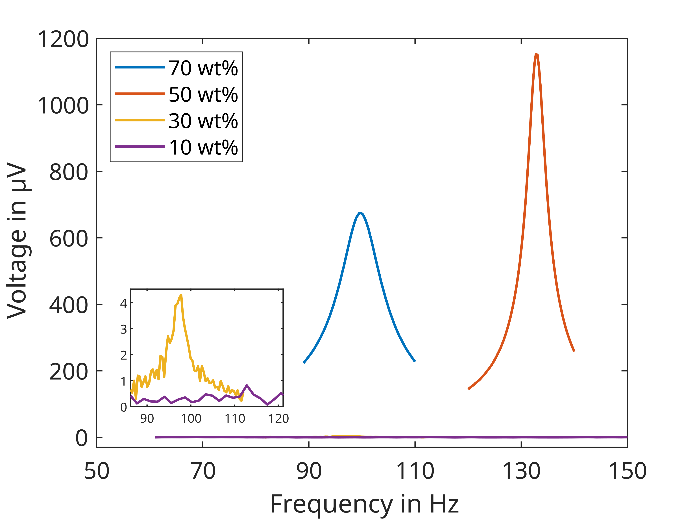


**Figure S1**: Amplitude response of the sensor elements with different particle concentrations. The sensor element with a 50 wt.% concentration generates the largest signal. The inset reveals the resonance frequency of the 30 wt.% sample. It also verifies that the 10 wt.% sensor element does not measure any magnetic field.

Figure 2S depicts the sensitivity measurements for the various sensor elements. The sensor with a particle concentration of 30 wt.% has a sensitivity of 4.3 µV at a field of 100 µT. The highest sensitivity of 1.15 mV at 100 µT is generated by the sensor with a 50 wt.% particle concentration. However, further increasing the particle filling factor to 70 wt.% results in a reduction in sensitivity to 0.67 mV at 100 µT.


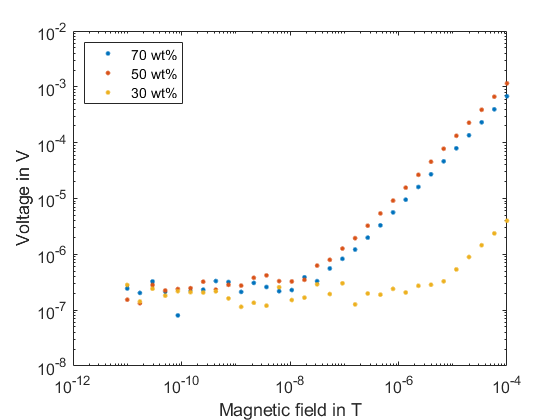


**Figure 2S**: Sensitivity measurements of the sensors with the MPCs with 30%, 50% and 70%, the sensors with only 10% are not considered because no magnetic fields could be detected. The noise floor is the same for all sensors.

The measurement involved applying a static (DC) bias field within the range of -1 mT to 1 mT. Starting at 1 mT, it increased in 0.1 mT increments until reaching 0 mT, then reversed to return from 0 mT to 1 mT (depicted as blue open circles) and all the way back to -1 mT (red crosses), cf. figure S3. The bias field does not change the magnetization of the sensor. Thus, the data points of the sweeps in both directions show the same value with a slight deviation originating from the accuracy of the measurement.

Between -200 µT and -300 µT there is a noticeable gap created by the measurement equipment, caused by a change of the measurement range. The voltage signal reaches a constant value for values larger than ± 600 µT. The largest signal is reached at a bias field of -533 µT.


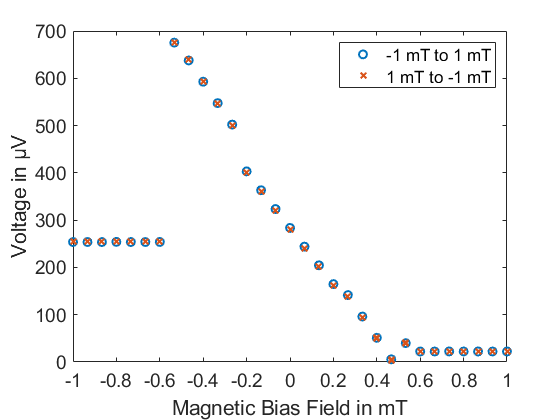


Figure S3: A bias measurement identified the optimal magnetic working point of the sensor. To ensure that the sensor's magnetization is not changed during the measurement, the bias field was first altered from ‑1 mT to 1 mT (black data points) and then the other way around (red data points). The magnetization of the sensor element is not changed, and the optimal working point is at ‑533 mT. The measurement was performed, while the sensor was excited with an AC field of 100 µT at the resonance frequency of 160.5 Hz.

In addition to the cantilevers, cylindrical samples with a diameter of 10 mm and a height of 25 mm were fabricated to evaluate the variation in mechanical properties with different compositions. Figure S4 presents the results for samples with varying filling factors (FF). The samples were subjected to compressive loading at different strain levels. Each compressive stress value represents the mean of 10 consecutive compressive tests. The compression levels applied were 2%, 10%, 20%, 40%, and 60%, demonstrating that the compressive stress increases with higher particle concentration. Consequently, samples with higher filling factor exhibit increased stiffness.


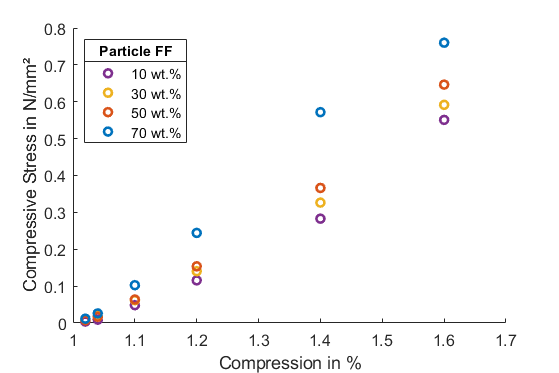


**Figure S4**: Summary of the compression measurements of the cylindrical samples with different filling factors (FF). Every data point is the mean of 10 consecutive measurements. With increasing filling factor, the stiffness of the sample increases as well.
